# Workplace assessment in crisis? The way forward

**DOI:** 10.1192/pb.bp.114.050153

**Published:** 2016-04

**Authors:** Denis O'Leary, Hasanan Al-Taiar, Nicholas Brown, Tomasz Bajorek, Marjan Ghazirad, Farshad Shaddel

**Affiliations:** 1 Royal College of Psychiatrists, London; 2 Health Education Thames Valley; 3 Oxford University

## Abstract

A recent Royal College of Physicians' study on assessment raises serious questions for workplace assessment. To address these, a system is recommended that bridges the gap from competence to performance and integrates supervised learning events (SLEs) that are formative in purpose with summative assessment of performance by entrustable professional activities (EPAs).

Workplace assessment is once again a matter for discussion, following the recent findings of a pilot study by the Royal College of Physicians (RCP).^1^ Acting on recommendations from a General Medical Council (GMC) working party on assessment,^2^ the RCP revised its assessment processes to differentiate between assessments of development and assessments of performance. The former are formative and aim to identify a trainee's areas of strength and development; the latter are summative and aim to determine fitness to progress. To underscore these differences in purpose the RCP adopted the terms supervised learning events (SLEs) and assessment of performance (AoP) proposed by the GMC. Of note is that the same workplace-based assessment (WPBA) tool can be used for each type of assessment; the assessment's purpose is the discriminating factor.

As a working group on assessment in psychiatry we were interested in the RCP findings, which represent a significant milestone in studies of workplace assessment. Although there are important methodological and interpretative limitations – e.g. small numbers of respondents to questionnaires and focus groups; their confusion regarding the purpose of assessments and how they inform a trainee's fitness to progress to their next stage of training (determined in the UK by Annual Review of Competency Progression (ACRP) panels) – arguably the findings reveal a crisis in postgraduate workplace assessment. To understand the implications for psychiatry, we summarise in this editorial the report's objectives, findings and recommendations. We then present proposals for the future direction of workplace assessment that integrate formative and summative assessment in the workplace in a way that builds on existing practice, educational theory and the study's findings.

## RCP study on workplace assessments: findings

The RCP aims were to evaluate the feasibility, validity and educational value of using existing WPBA tools but for different assessment purposes (formative and summative) and processes, i.e. SLEs and AoP. SLE judgements purposefully excluded any numerical descriptors in order to focus on their formative purpose (feedback, reflection and action-planning); AoP were judged as pass/fail in keeping with their summative purpose. The study commenced in August 2012 for a period of one academic year across three Deaneries and included all trainees (546 core, 309 higher) and 669 trainers across nine medical specialties. A mixed-method qualitative approach was used to generate triangulated findings from responses to a series of questionnaires and focus groups.

In terms of educational value, it is important to note the study's finding that neither trainees nor trainers considered WPBAs to be of educational value before participating in the revised system. They subsequently responded that in the new approach SLEs, but not AoPs, had some educational value, although as noted above their purpose and differences between them were understood poorly. Adherence with the prescribed conduct of WPBA tools was variable (e.g. observational tools were rated retrospectively and without direct trainer observation) and even for SLEs their educational gain was limited by the lack, delay and poor quality of feedback and action-planning. Undertaking SLEs and AoPs was feasible, although there were work and time constraints. Additional constraints pertained to trainer engagement across each type of assessment and trainee engagement with AoPs. SLEs were considered to have construct validity, but AoPs were not – only 1% of AoP assessments judged a performance as below the standard expected. Such low rates of recorded underperformance were attributed to poor definition of the required standards and reluctance by trainers to risk potential trainee confrontation. The report recommends that AoPs as used in the study be discontinued but that SLEs continue alongside recommendations for improved training on the purpose and conduct of WPBAs, feedback and action-planning, and consideration of their inclusion in the ARCP process.

## Implications for psychiatry

What are the implications for psychiatry, where similar WPBA tools are used, although not in the way recommended by the GMC? Are we to abandon WPBAs to inform assessment of performance? Where does that leave the reliability and validity of decisions on training progression? How can levels of trainer engagement and the training value of assessment be improved? It is our view that an assessment system that integrates SLEs and ‘entrustable professional activities’ (EPAs)^3,4^ will help address not only the issue of summative (‘high stakes’) judgements on performance but the wider issues cited here as well. This statement requires further elaboration, beginning with a description of EPAs.

## Entrustable professional activities

An EPA is a core unit of work essential to clinical practice and performed to a specified level of entrustability or clinical supervision, for instance a new out-patient assessment presented to the clinical supervisor over the telephone (distant supervision). These are new in practice, although used in the USA across several specialties (family and internal medicine, paediatrics) and undergraduate programmes.^5^ More significantly for psychiatry, they have been developed and incorporated into curricular design and training programmes by the Royal Australian and New Zealand College of Psychiatry (RANZCP).^6^

As well as a clear title and description of the work and context, EPA content is underpinned by a competency framework (and curriculum) and as such can be supported by formative assessment using specific SLEs and existing WPBAs. Although necessary and in keeping with SLEs' formative purpose, when making a judgement on an EPA the outcome of these SLE assessments is integrated with other evidence, formal and informal, about a trainee's workplace performance on this unit of work. As such, the EPA/summative judgements, triangulated across several sources of evidence, can contribute to ARCP/progression decisions. Additionally, as the EPA criteria specify increasing levels of entrustability (decreased need for supervision), they can be used to demonstrate performance over time towards the expected and growing level of independent practice – or distant supervision for trainees. Such ‘progressive independence in training’^7^ is facilitated by clinical and educational supervisors, and improved trainee confidence, motivation and identity as a psychiatrist are considered best practice in experience-based learning.^8^ Such improved proficiency is included by Harden^9^ as one of four ‘exit learning outcomes’, alongside increased clinical depth, breadth and utility of application.

## Advantages of the proposed system

Although it is likely that these proposed changes to the assessment process will improve the formative value of WPBA, the key questions are, will they improve the level of trainer and trainee engagement in workplace assessment of performance and judgements on whether performance meets satisfactory standards. We believe they can as in terms of engagement:

- initial design of the requisite EPAs requires value judgements by trainers and trainees alike on what represent core units of work
- the approach focuses the use of WPBAs as formative tools with self-assessment and reflection input by the trainee, feedback from the clinical supervisor and joint action-planning^10^
- inclusion of the required level of supervision as part of SLE feedback enables future decisions on meeting performance standards
- the outcome of SLEs can be included in documentation for the ARCP panel (as suggested by trainees in the survey)^1^
- the requirement that the outcomes of specific SLEs are used to inform judgements on specific EPA sign-off will focus assessment on key units of professional activity underpinned by the curriculum, thus moving away from current requirements based on numbers of WPBAs alone
- EPAs, being triangulated, can contribute directly and more meaningfully to ARCP progression decisions
- the approach is likely to be more meaningful to both trainees and trainers and is consistent with educational models of postgraduate training that emphasise the role of the clinical supervisor as facilitator and the trainee as part of a community of practice.^9^

The process will enable judgement on performance standards through the:

- use of several sources of information (increasing triangulation) rather than the outcome of a single workplace assessment – the outcomes of specific SLEs are necessary but not sufficient;
- the addition of a ‘level of required supervision’ criterion will support EPA judgements and places patient safety at the heart of the process;
- generally, although WPBA may not identify underperformance at the same prevalence as formal examination testing, nevertheless it is reasonable to expect that when it does judge underperformance it is identifying a population at high risk of poor performance across testing generally.

While being mindful of the multi-determined nature of assessment and the reality that any new system aims for improvement rather than perfection, we conclude that the Royal College of Psychiatrists should pilot a revised assessment system that: (i) adopts the GMC's recommendation on types of WPBA; (ii) aligns SLEs (formative assessments) that are conducted against a competency framework using existing WPBA tools modified to improve the quality of reflection (self-assessment on performance), feedback and action-planning and to provide a statement on the level of ongoing clinical supervision required; and (iii) enables the outcome of a specified number of targeted SLEs to contribute to summative judgements by educational supervisors on trainee performance across specified EPAs. The pilot should test the system's feasibility, validity and educational impact and the findings considered alongside any evaluation from the RANZCP process. Without such an initiative a crisis of confidence in workplace assessment will serve only to erode best educational practice.
